# Suboptimal medical care of patients with ST-Elevation Myocardial Infarction and Renal Insufficiency: results from the Korea acute Myocardial Infarction Registry

**DOI:** 10.1186/1471-2369-13-110

**Published:** 2012-09-11

**Authors:** Joon Seok Choi, Chang Seong Kim, Eun Hui Bae, Seong Kwon Ma, Myung Ho Jeong, Young Jo Kim, Myeong Chan Cho, Chong Jin Kim, Soo Wan Kim

**Affiliations:** 1 Department of Internal Medicine, Chonnam National University Medical School, 42 Jebongro, Gwangju, 501-757, South Korea; 2 Cardiovascular Research Institute of Chonnam National University, Gwangju, South Korea; 3 Department of Internal Medicine, Yeungnam University, Daegu, South Korea; 4 Department of Internal Medicine, Chungbuk National University, Cheongju, South Korea; 5 Department of Internal Medicine, Kyunghee University, Seoul, South Korea

**Keywords:** Myocardial infarction, Optimal medical care, Renal function

## Abstract

**Background:**

The clinical outcomes of ST-segment elevation myocardial infarction (STEMI) are poor in patients with renal insufficiency. This study investigated changes in the likelihood that patients received optimal medical care throughout the entire process of myocardial infarction management, on the basis of their glomerular filtration rate (GFR).

**Methods:**

This study analyzed 7,679 patients (age, 63 ± 13 years; men 73.6%) who had STEMI and were enrolled in the Korea Acute Myocardial Infarction Registry (KAMIR) from November 2005 to August 2008. The study subjects were divided into 5 groups corresponding to strata used to define chronic kidney disease stages.

**Results:**

Patients with lower GFR were less likely to present with typical chest pain. The average symptom-to-door time, door-to-balloon time, and symptom-to-balloon time were longer with lower GFR than higher GFR. Primary reperfusion therapy was performed less frequently and the results of reperfusion therapy were poorer in patients with renal insufficiency; these patients were less likely to receive adjunctive medical treatment, such as treatment with aspirin, clopidogrel, β-blocker, angiotensin-converting enzyme (ACE) inhibitor/angiotensin-receptor blocker (ARB), or statin, during hospitalization and at discharge. Patients who received less intense medical therapy had worse clinical outcomes than those who received more intense medical therapy.

**Conclusions:**

Patients with STEMI and renal insufficiency had less chance of receiving optimal medical care throughout the entire process of MI management, which may contribute to worse outcomes in these patients.

## Background

Acute coronary syndrome (ACS) is the leading cause of death in patients with chronic kidney disease (CKD) [1,2]. The severity of renal insufficiency, including mild to moderate renal insufficiency is directly associated with increased cardiovascular morbidity and mortality [3,4]. Several factors may be related to poorer outcomes of ACS in patients with renal insufficiency. Hypertension, dyslipidemia, and diabetes mellitus (DM) are common among patients with renal insufficiency and are often inadequately treated in this population [5]. In addition, the accumulation of uremic toxins can stimulate oxidative stress and inflammation, and hence, contribute to endothelial dysfunction and atherosclerosis progression [6].

Primary management in patients with ST-segment elevation myocardial infarction (STEMI) includes timely restoration of flow in the stenosed artery—either by fibrinolysis or by percutaneous coronary intervention (PCI)—to limit the extent of infarction in the myocardium. In addition, medications (anti-platelet agent, β-blocker, angiotensin-converting enzyme [ACE] inhibitor or angiotensin-receptor blocker [ARB] and statin) form an important component of evidence-based management. These management strategies have contributed to improved survival in patients with STEMI. However, only a few studies have evaluated how renal function affects the likelihood of receiving optimal medical care throughout the entire treatment period in patients with STEMI.

The purpose of this study was to investigate whether renal insufficiency is associated with a decreased likelihood of receiving optimal medical care in patients with STEMI. The entire medical treatment process for patients with STEMI was comprehensively analyzed, and the relationship between optimal management and clinical outcomes in patients with renal insufficiency was investigated.

## Methods

### Korea acute myocardial infarction registry

The study population was derived from the Korea Acute Myocardial Infarction Registry (KAMIR). The KAMIR is a prospective, open, observational, multicenter online registry investigating the risk factors for mortality in patients with acute myocardial infarction (AMI) in Korea since November 2005. Through the support of the Korean Circulation Society, 52 community and university hospitals that had facilities for primary PCI and sufficient experience with the procedure participated in KAMIR with the aim of establishing clinical practice guidelines for AMI. The name of each participation centers listed in the Appendix 1 and 2. Data were collected at each site by a trained study coordinator by using a standardized protocol. The study protocol was approved by the ethics committee at each participating institution. All patients gave written informed consent before enrollment.

### Study population

This study retrospectively analyzed a cohort of 7,679 consecutive patients who were admitted to the hospital between November 1, 2005, and July 31, 2008, and had a discharge diagnosis of STEMI, confirmed by both cardiac enzyme and electrocardiogram analyses. The diagnosis of STEMI was based on a suggestive history, with ST elevation > 2 mm in ≥2 precordial leads, ST elevation > 1 mm in ≥2 limb leads, or new left branch bundle block on the 12-lead electrocardiogram with a concomitant increase of cardiac markers ≥2 times the upper limit of normal. Patients were excluded if an estimated glomerular filtration rate (GFR) could not be calculated.

Renal function was based on GFR estimation. The Chronic Kidney Disease Epidemiology Collaboration (CKD-EPI) equation was used to estimate GFR in milliliters per minute per 1.73 m^2^[7]. The study population was divided into 5 groups according to the estimated GFR: group I, GFR ≥ 90 mL/min/1.73 m^2^ (2099 patients; age, 54 ± 11 years; men, 83.1%); group II, 60 ≤ GFR < 90 mL/min/1.73 m^2^ (3647 patients; age, 63 ± 12 years; men, 75.8%); group III, 30 ≤ GFR < 60 mL/min/1.73 m^2^ (1600 patients; age, 71 ± 11 years; men, 60.6%); group IV, 15 ≤ GFR < 30 mL/min/1.73 m^2^ (210 patients; age, 73 ± 11 years; men, 45.2%); and group V, GFR < 15 mL/min/1.73 m^2^ (123 patients; age, 66 ± 13 years; men, 64.2%).

### Data collection

The baseline variables included age; gender; body mass index (BMI); and several coronary risk factors such as hypertension (defined as history of hypertension and admission blood pressure > 140 mm Hg systolic or > 90 mm Hg diastolic), DM (defined as history of DM or random blood glucose level > 200 mg/dL), hyperlipidemia (defined as history of hyperlipidemia, total cholesterol level of > 240 mg/dL, or low density lipoprotein [LDL] level > 101 mg/dL), history of smoking, history of ischemic heart disease (IHD), clinical symptoms at admission (chest pain or dyspnea), and Killip class. One of the following 3 modalities was selected as primary reperfusion therapy for patients with STEMI: intravenous thrombolytic therapy, primary PCI, or facilitated PCI. The use of certain medications (aspirin, clopidogrel, ACE inhibitor, ARB, β-blocker or statin) during the in-hospital period and at discharge was also recorded. MACE was defined as a composite outcome of cardiac death, repeated PCI, or MI.

### Statistical analysis

Continuous variables with normal distributions are expressed as mean ± SD, and the 5 groups were compared using one-way ANOVA. Continuous data with a skewed distribution are presented as median (with 25th and 75th percentiles) and were compared using the Kruskal-Wallis test. Categorical variables were compared using the chi-square test or the Fisher’s exact test if the expected value of the variable was <5 in at least 1 group. Survival analysis after MI was estimated using the Kaplan-Meier method with log-rank tests to compare survival among groups. All statistical tests were 2-tailed, and p < 0.05 was considered significant. Analyses were performed using the Statistical Package for Social Sciences software, version 18.0 (IBM, Armonk, NY USA).

## Results

### Baseline characteristics

A total of 7,679 patients (age, 63 ± 13 years; men, 73.6%) were included in the present study. The baseline characteristics and biochemical parameters of patients are shown in Table1. A lower GFR was associated with older age, female gender, higher prevalence of hypertension, DM, history of IHD, and lower likelihood of being a current smoker. At the time of hospital arrival, a lower GFR was associated with lower blood pressure, lower left ventricular ejection fraction (LVEF), and higher Killip class. In patients with lower GFR, the total cholesterol, LDL, triglyceride, troponin-I, and creatine kinase-MB (CK-MB) levels were lower, whereas the glucose, high sensitivity C-reactive protein (hs-CRP), N-terminal prohormone of brain natriuretic peptide (NT-pro BNP) levels were higher than in patients with higher GFR.

**Table 1:** Baseline characteristics

	**Group I (n = 2099)**	**Group II (n = 3647)**	**Group III (n = 1600)**	**Group IV (n = 210)**	**Group V (n = 123)**	***P*value**
Age, years	54 ± 11	63 ± 12	71 ± 11	73 ± 11	66 ± 13	<0.001
Male	1745(83.1)	2763(75.8)	970(60.6)	95(45.2)	79(64.2)	<0.001
BMI, kg/m^2^	24 ± 3	24 ± 3	24 ± 3	23 ± 3	24 ± 3	0.284
**Past History**						
Hypertension	739(35.3)	1571(43.2)	953(59.8)	156(74.6)	75(61.5)	<0.001
DM	445(21.3)	789(21.7)	505(31.7)	99(47.6)	60(48.8)	<0.001
Previous IHD	190(9.1)	420(11.6)	239(15.1)	39(18.8)	29(23.6)	<0.001
Hyperlipidemia	184(8.8)	273(7.5)	121(7.6)	17(8.1)	8(6.6)	0.171
Smoking	1276(61.1)	1779(49.1)	493(31.2)	53(25.9)	39(32.2)	<0.001
**At admission**						
SBP (mmHg)	130 ± 26	127 ± 28	117 ± 33	111 ± 36	123 ± 35	<0.001
DBP (mmHg)	81 ± 16	78 ± 17	72 ± 19	70 ± 23	75 ± 21	<0.001
Killip class	1.3 ± 0.7	1.4 ± 0.8	1.8 ± 1.1	2.1 ± 1.2	2.0 ± 1.2	<0.001
LVEF (%)	52 ± 11	51 ± 12	48 ± 13	47 ± 14	47 ± 12	<0.001
**Biochemical parameters**						
Creatinine (mg/dL)	0.8 ± 0.1	1.0 ± 0.2	1.4 ± 0.3	2.3 ± 0.5	9.5 ± 8.9	<0.001
Troponin-I (ng/mL)	40(9,79)	30(7,68)	28(5,73)	31(10,56)	28(6,51)	0.007
CK-MB (U/L)	133(38,277)	132(40,283)	112(26,243)	84(27,195)	69(20,176)	<0.001
TC (mg/dL)	188 ± 44	183 ± 43	175 ± 45	165 ± 42	174 ± 60	0.004
LDL (mg/dL)	122 ± 42	116 ± 42	112 ± 41	100 ± 39	106 ± 52	0.026
TG (mg/dL)	109(72,163)	98(66,143)	95(66,140)	105(74,152)	91(66,150)	<0.001
Glucose (mg/dL)	161 ± 66	168 ± 70	201 ± 100	234 ± 134	201 ± 106	<0.001
hs-CRP (mg/dL)	0.6(0.2,3.4)	0.8(0.2,4.2)	1.3(0.3,7.0)	4.6(0.8,15.0)	2.1(0.6,9.8)	<0.001
NT-proBNP (pg/mL)	223 (57,871)	346 (75,1414)	1144 (230,3973)	5423 (1296,21374)	21191 (4957,35000)	<0.001

Continuous data are expressed as the mean ± SD or the median and interquartile range (25th and 75th percentiles). Categorical data were expressed as percentages. Abbreviations: BMI, body mass index; DM, diabetes mellitus; IHD, ischemic heart disease; SBP, systolic blood pressure; DBP, diastolic blood pressure; LVEF, left ventricular ejection fraction; CK-MB, creatine kinase-MB; TC, total cholesterol; LDL, low density lipoprotein; hs-CRP, high-sensitivity C-reactive protein; NT-proBNP, N-terminal pro B-type natriuretic peptide.

### Delayed timely restoration of flow in the stenosed artery

Patients with lower GFRs were less likely to present with typical chest pain. The time to restoration of flow in the stenosed artery was analyzed in the following ways: symptom-to-door time in all patients; door-to-balloon time and symptom-to-balloon time in patients who underwent primary PCI as initial reperfusion therapy; door-to-needle time and symptom-to-needle time in patients who received thrombolysis as initial reperfusion therapy (Table2). Patients with lower GFRs tended to have longer symptom-to-door times. In patients with lower GFRs, door-to-balloon time and symptom-to-balloon time also tended to be longer when primary PCI was the initial reperfusion therapy. In contrast, door-to-needle time and symptom-to-needle time were not significantly different in patients with lower GFRs.

**Table 2:** Presence of typical chest pain and time of restoration of infarcted-artery

	**Group I**	**Group II**	**Group III**	**Group IV**	**Group V**	***P*value**
Presence of chest pain	1916(91.8)	3215(88.7)	1305(82.2)	154(74.4)	80(66.1)	<0.001
Symptom to door time, hr	3.5(1.8,7.7)	3.3(1.6,8.2)	3.8(1.8,9.7)	5.4(2.1,15.5)	4.8(1.8,16.1)	<0.001
**Primary PCI** †						
Door to ballon time, hr	1.5(1.1,2.9)	1.5(1.0,2.3)	1.5(1.1,2.4)	1.7(1.2,2.6)	1.9(1.3,4.6)	<0.001
Symptom to ballon time, hr	5.6(3.4,13.0)	4.8(3.1,10.1)	5.4(3.3,10.8)	8.3(4.2,18.4)	7.2(3.3,26.0)	<0.001
**Thrombolysis** ‡						
Door to needle time, hr	0.9(0.6,1.5)	0.9(0.6,1.4)	0.9(0.6,1.4)	1.2(0.4,5.8)	0.8(0.4,-)	0.876
Symptom to needle time, hr	3.5(2.2,5.0)	3.4(2.4,5.1)	3.4(2.4,5.1)	5.0(4.1,9.0)	11.0(0.8,-)	0.316

Continuous data are expressed as the median and interquartile range (25th and 75th percentiles). Categorical data were expressed as percentages. Abbreviations: PCI, percutaneous intervenyion;

† The subject who received primary reperfusion therapy as primary percutaneous intervention (PCI), (n = 5,607).

‡ The subject who received primary reperfusion therapy as thrombolytic therapy, (n = 618).

### Initial treatment strategy and results of reperfusion therapy

**Figure 1:**
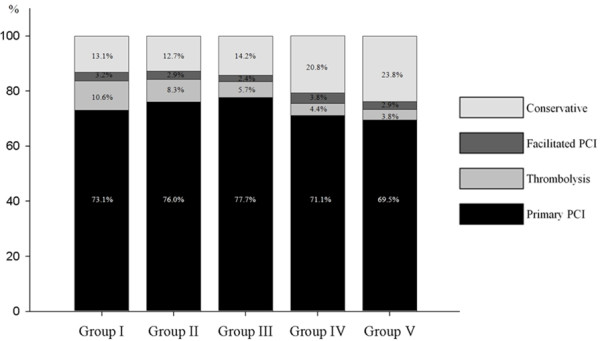
Initial reperfusion strategy in STEMI patients.

In the initial selection of treatment strategy in patients with STEMI, conservative treatment was more commonly performed but thrombolysis was less frequently performed in patients with lower GFRs (Figure1). During the in-hospital period, PCI, regardless of its subtype (primary PCI, facilitated PCI, or rescue PCI), was less frequently performed in patients with lower GFR, and its success rate markedly decreased with decrease in GFR (Table3). In addition, thrombolysis was also performed less frequently in patients with lower GFR, and the success rate of thrombolysis decreased with decrease in GFR.

**Table 3:** Results of reperfusion therapy

	**Group I**	**Group II**	**Group III**	**Group IV**	**Group V**	***P*value**
**Result of PCI** †						
Patients, n	1974(95.9)	3406(94.9)	1437(91.3)	181(87.4)	104(86.0)	<0.001
Success	1832(92.8)	3139(92.2)	1286(89.5)	151(83.4)	86(82.7)	<0.001
Suboptimal	40(2.0)	83(2.3)	44(3.1)	6(3.3)	5(4.8)	
Failed	102(5.2)	184(5.4)	107(7.4)	24(13.3)	13(12.5)	
**Result of thrombolysis** ‡						
Patients, n	290(14.0)	367(10.2)	119(7.5)	11(5.3)	6(5.0)	<0.001
Success	222(76.6)	262(71.4)	83(69.7)	3(27.3)	3(50.0)	0.003
Failed	68(23.4)	105(28.6)	36(30.3)	8(72.7)	3(50.0)	

Data are presented as number of patients (percentage). Abbreviations: PCI, percutaneous intervention.

† The subject who received PCI during in-hospital period regardless of its subtype (primary PCI, facilitated PCI, rescue PCI), (n = 7,102).

‡ The subject who received thrombolysis therapy during in-hospital period (thrombolysis only, facilitated PCI), (n = 793).

### Angiographic finding

Angiographic findings are shown in Table4. Multivessel coronary artery disease and disease of the left main coronary artery were more common in groups with lower GFR. Initial TIMI flow 0 (no flow) and presence of a complex lesion (type C) were more frequently observed and a final TIMI flow 3 (complete perfusion) was less frequently observed with lower GFR. Patients with lower GFR were treated with a greater number of stents than patients with higher GFRs.

**Table 4:** Angiographic data

	**Group I**	**Group II**	**Group III**	**Group IV**	**Group V**	***P*value**
**Number of vessel**						
One vessel	1044(53.2)	1599(47.2)	508(35.6)	42(23.3)	31(30.1)	<0.001
Two vessel	527(26.9)	1035(30.6)	466(32.7)	66(36.7)	30(29.1)	<0.001
Three vessel	354(18.1)	682(20.1)	405(28.4)	62(34.4)	35(34.0)	<0.001
LM	36(1.8)	70(2.1)	47(3.3)	10(5.6)	7(6.8)	<0.001
ACC/AHA lesion C	903(48.9)	1625(51.4)	714(53.8)	108(65.5)	57(59.4)	<0.001
Initial TIMI flow grade 0	938(49.7)	1763(54.1)	772(56.1)	102(59.3)	41(41.8)	0.007
Final TIMI flow grade 3	1728(94.3)	2913(91.6)	1181(88.1)	139(84.2)	76(81.7)	<0.001
Stent number	1.40 ± 0.74	1.44 ± 0.77	1.48 ± 0.81	1.49 ± 0.75	1.52 ± 0.88	0.002
Stent diameter, mm	3.21 ± 0.42	3.21 ± 0.44	3.15 ± 0.45	3.10 ± 0.43	3.22 ± 0.42	0.655
Stent length, mm	25.0 ± 6.3	25.1 ± 6.2	25.3 ± 6.4	26.4 ± 6.8	24.7 ± 6.5	0.802

Data are presented as mean ± SD or number of patients (percentage). Abbreviations: TIMI, Thrombolysis In Myocardial Infarction.

### Intensity of medical treatment

**Figure 2:**
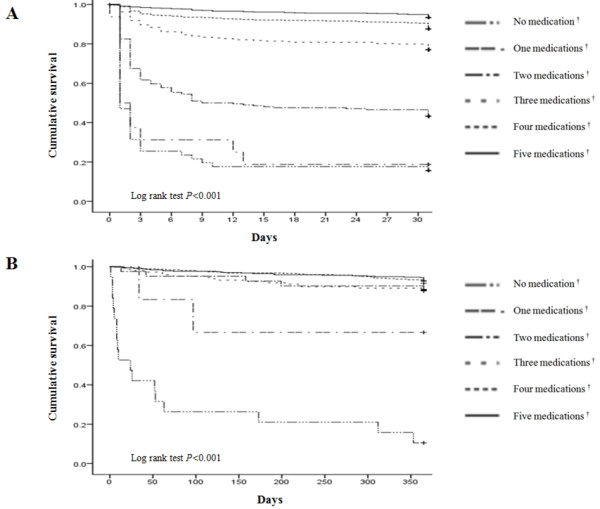
**Kaplan-Meier estimates of cumulative survival according to intense of medical therapies.** (**A**) 1 month cumulative survival according to intense of in-hospital medications (**B**) 1 year cumulative survival according to intense of discharge medications; medications^†^, sum of prescription of aspirin, clopidogrel, beta-blocker, angiotensin converting enzyme inhibitor or angiotensin receptor blocker and statin.

Table5 lists specific medical treatments received prior to PCI, both during the in-hospital period and at discharge, according to GFR. The use of an anti-platelet agent prior to PCI was not significantly different among the groups. During in-hospital periods, standard medications known to improve survival after MI, such as anti-platelet agent, β-blocker, ACE inhibitor or ARB, and statin were less frequently used in patients with lower GFR. The underuse of these drugs was not corrected until hospital discharge of the patients who survived STEMI. We evaluated the correlation between clinical outcomes and intensity of evidence-based medication in patients with GFR < 60 mL/min/1.73 m^2^. In the hospital period, the patients who received less intense medical therapies had worse clinical outcomes than those who received more intense medical therapies. Similar observations were made for discharge medications among patients who survived STEMI (Figure2).

**Table 5:** Intense of medical therapy

	**Group I**	**Group II**	**Group III**	**Group IV**	**Group V**	***P*value**
**Pre-PCI medication**						
Patients, n	1974	3406	1437	181	104	96
Aspirin	796(40.3)	1366(40.1)	565(39.3)	76(42.0)	53(51.0)	0.476
Clopidogrel	748(37.9)	1299(38.1)	528(36.7)	71(39.2)	49(47.1)	0.622
**In-hospital medication**						
Patients, n	2099	3647	1600	210	123	
Aspirin	2069(98.6)	3594(98.5)	1544(96.5)	197(93.8)	118(95.9)	<0.001
Clopidogrel	2048(97.6)	3540(97.1)	1522(95.1)	192(91.4)	114(92.7)	<0.001
β-blocker	1619(77.1)	2581(70.8)	1003(62.7)	115(54.8)	71(57.7)	<0.001
ACEi or ARB	1754(83.6)	2906(79.7)	1165(72.8)	132(62.9)	73(59.3)	<0.001
Statin	1630(77.7)	2716(74.5)	1034(64.6)	125(59.5)	79(64.2)	<0.001
**Discharge medication**						
Patients, n	2067	3519	1373	151	91	
Aspirin	1980(95.8)	3390(96.3)	1303(94.9)	139(92.1)	86(94.5)	0.054
Clopidogrel	1927(93.2)	3276(93.1)	1266(92.2)	131(86.8)	81(89.0)	0.012
β-blocker	1528(73.9)	2412(68.5)	921(67.1)	99(65.6)	58(63.7)	<0.001
ACEi or ARB	1691(81.8)	2789(79.3)	1070(77.9)	105(69.5)	66(72.5)	<0.001
Statin	1577(76.3)	2610(74.2)	941(68.5)	101(66.9)	61(67.0)	<0.001

Data are presented as number of patients (percentage). Abbreviations: PCI, percutaneous coronary intervention; ACEi, angiotensin converting enzyme inhibitor; ARB, angiotensin receptor blocker.

### Outcomes according to GFR

The clinical outcomes in patients with STEMI are listed in Table6. At 1 month, MACE and all-cause death were higher in patients with lower GFRs. At 12 months, MACE and all-cause death also increased as decreasing GFRs.

**Table 6:** Outcomes according to the GFR

	**Group I**	**Group II**	**Group III**	**Group IV**	**Group V**	***P*value**
**1 month outcomes**						
MACE	64(3.5)	217(6.9)	284(20.6)	74(41.8)	37(35.2)	<0.001
All-cause death	41(2.0)	160(4.4)	263(16.4)	71(33.8)	35(28.5)	<0.001
**12 month outcomes**						
MACE	183(8.7)	382(10.5)	351(21.9)	78(37.1)	46(37.4)	<0.001
All-cause death	60(4.4)	195(8.2)	304(27.1)	79(54.9)	41(48.8)	<0.001

Data are presented as number of patients (percentage). Abbreviations: MACE, major adverse cardiovascular event.

## Discussions

The principal finding of this study is that patients who have renal insufficiency and develop STEMI are less likely to receive optimal therapy. Patients with renal insufficiency more often presented with atypical symptoms and had longer delays in both hospital arrival and reperfusion. In addition, primary reperfusion therapy was performed less frequently in patients with renal insufficiency and the results of reperfusion therapy were worse with lower GFR. Patients with renal insufficiency were less likely to receive life-saving medical medications such as anti-platelet agent, β-blocker, ACE inhibitor/ARB, or statin during hospitalization and at discharge.

A recent study reported that the probability of patients with MI presenting without chest pain increased proportionally with a decrease in GFR [8]. Canto et al. also showed that patients with MI, but without chest pain, are at increased risk for delays in seeking medical attention, less aggressive treatment, and have higher associated in-hospital mortality [9]. Taken together, these findings suggest that patient with renal insufficiency may have less chance of receiving timely medical care, which may in part be attributed to lack of chest pain. A delay in the time to restoration of flow in the stenosed artery was also observed with lower GFR in patients who had STEMI and underwent primary PCI. Rapid reperfusion is an important goal in the treatment of STEMI patients [10,11]. However, patients with renal insufficiency arrived at the hospital relatively late with respect to symptom onset, and clinicians may be reluctant to make an immediate decision in patients with renal insufficiency because of the presence of more comorbidities and increased complexity of medical care.

During the hospital period, reperfusion therapy, regardless of its subtype, was used less frequently in patients with lower GFR, which was consistent with previous studies [12,13]. Older age, more comorbidities, compromised hemodynamic status, and fear of complications such as bleeding and contrast-induced nephropathy may all affect a clinician’s decision. In addition, the results of reperfusion therapy were disappointing in patients with renal insufficiency. A higher incidence of multivessel disease, more complex lesions, and poor coronary blood flow before and after PCI may contribute to the poor results of reperfusion therapy. Although the exact pathophysiologic mechanisms by which renal insufficiency adversely affect the results of reperfusion therapy have not been clearly elucidated, oxidative stress, endothelial cell dysfunction, and more advanced atherosclerosis may play a role in this regard [14-17].

Although, renal insufficiency is an important risk factor for increased mortality, patients with lower GFR were less likely to receive adjunctive medical therapies recommended by current guidelines. This finding is consistent with previous reports [18,19]. The underuse of life-saving medications may result from fear of adverse effects. Clinicians are often reluctant to use an anti-platelet agent for patients with renal insufficiency because of the risk of bleeding. Similarly, hyperkalemia or renal function deterioration often pushes clinicians to withhold ACE inhibitor/ARB therapy. However, evidence indicates that these medications are associated with great survival benefits in patients with renal insufficiency, even in the case of severe renal insufficiency [20-22]. In clinical practice, clinicians who assess the risks and benefits of medication should consider the fact that the long-term cardioprotective and survival benefits outweigh the risk associated with short-term adverse effects.

The mortality rate associated with MI has fallen considerably in recent decades [23,24]. Improvements in emergency system response times and diagnostic techniques may explain some of the improvements in MI mortality. Advances in medical treatment and cardiac interventions have also played a role in this improvement. Despite these advances, data from this study indicate that patients with renal insufficiency were less likely to receive optimal medical care throughout the entire process of MI management and that these differences may contribute to worse outcomes in patients from the KAMIR data population who had lower GFR and developed MI [4,25].

The present study has some limitations. First, KAMIR is a multicenter retrospective registry study and not a randomized controlled study. Therefore, unmeasured factors in the KAMIR database could influence these findings. Second, assessment of kidney function in this study was based on a single serum creatinine value obtained at the time of presentation to the hospital. This value may have been affected by hemodynamic or metabolic status. Third, although, estimating GFR based on serum creatinine are preferable method for accessing renal function in the clinical practice, it is influenced by various factors such as age, gender, ethnics, muscle mass and nutritional status. This could confound the relationship between GFR and likelihood of receiving optimal medical care in patients with STEMI.

## Conclusion

In conclusion, patients with STEMI and renal insufficiency had significantly less chance of receiving optimal medical care throughout the entire process of MI management. Despite recent advances in emergency systems and evidence-based therapy, these advances have been applied less frequently in patients with renal insufficiency than in patients with normal renal function. Therefore, patients with renal insufficiency require an aggressive approach to diagnosis, intervention, and medical treatment for improved clinical outcomes.

## Appendix 1

Korea Acute Myocardial infarction Registry (KAMIR) Investigators

Myung Ho Jeong MD, Young Keun Ahn MD, Sung Chull Chae MD, Jong Hyun Kim MD, Seung Ho Hur MD, Young Jo Kim MD, In Whan Seong MD, Dong Hoon Choi MD, Jei Keon Chae MD, Taek Jong Hong MD, Jae Young Rhew MD, Doo Il Kim MD, In Ho Chae MD, Jung Han Yoon MD, Bon Kwon Koo MD, Byung Ok Kim MD, Myoung Yong Lee MD, Kee Sik Kim MD, Jin Yong Hwang MD, Myeong Chan Cho MD, Seok Kyu Oh MD, Nae Hee Lee MD, Kyoung Tae Jeong MD, Seung Jea Tahk MD, Jang Ho Bae MD, Seung Woon Rha MD, Keum Soo Park MD, Chong Jin Kim MD, Kyoo Rok Han MD, Tae Hoon Ahn MD, Moo Hyun Kim MD, Ki Bae Seung MD, Wook Sung Chung MD, Ju Young Yang MD, Chong Yun Rhim MD, Hyeon Cheol Gwon MD, Seong Wook Park MD, Young Youp Koh MD, Seung Jae Joo MD, Soo Joong Kim MD, Dong Kyu Jin MD, Jin Man Cho MD, Byung Ok Kim MD, Sang-Wook Kim MD, Jeong Kyung Kim MD, Tae Ik Kim MD, Deug Young Nah MD, Si Hoon Park MD, Sang Hyun Lee MD, Seung Uk Lee MD, Hang-Jae Chung MD, Jang Hyun Cho MD, Seung Won Jin, MD, Yang Soo Jang MD, Jeong Gwan Cho, MD and Seung Jung Park MD.

## Appendix 2

Korea Acute Myocardial infarction Registry (KAMIR) participation centers

Seoul St.Mary's Hospital, Kangdong Sacred Hospital, Konyang University Hospital, Kyungpook National University Hospital, Gyeongsang National University Hospital, Kyunghee Dongsuh University Hospital, Kyunghee University Medical Center, Korea University Guro Hospital, Kwangju Christian Hospital, National Health Insurance Corporation Ilsan Hospital, Dankook University Hospital, Daegu Catholic University Medical Center, Daejeon Sun Hospital, Daejeon St.Mary's Hospital, Dongguk University Gyeongju Hospital, Dongsan Medical Center, Dong-A University Medical Center, Mokpo Hankuk Hospital, Kwangju Veterans Hospital, Busan National University Hospital, Busan Marynoll Medical Center, Busan Paik Hospital, Busan Hanseo Hospital, Seoul National University Bundang Hospital, Samsung Medical Center, Inje University Sanggye Paik Hospital, Seoul National University Hospital, Asan Medical Center, St. Paul's Hospital, St. Carollo Hospital, Soon Chun Hyang University Bucheon Hospital, Soon Chun Hyang University Cheonan Hospital, Yonsei University Severance Hospital, Ajou University Hospital, Yeouido St.Mary's Hospital, Yeungnam University Medical Center, Wonkwang University School of Medicine & Hospital, Wonju University Hospital, Eulji Medical Center, Ewha Womans University Mokdong Hospital, Gachon University Gil Hospital, Inha University Hospital, Chonnam National University Hospital, Chonbuk National University Hospital, Presbyterian Medical Center, Jeju National University Hospital, Chosun University Hospital, Chung Ang University Hospital, Chungnam National University Hospital, Chungbuk National University Hospital, Pohang Seomyung Christianity Hospital, Hallym University Medical Center.

## Competing interests

The authors declared that they have no competing interest.

## Authors’ contributions

JSC and SWK designed research, performed, and wrote the paper. CSK, EHB and SKM interpreted the data. MHJ, YJK, MCC and CJK were investigators of Korea Acute Myocardial infarction Registry. All authors read and approved the final manuscript.

## Pre-publication history

The pre-publication history for this paper can be accessed here:

http://www.biomedcentral.com/1471-2369/13/110/prepub
